# Magnetism in monolayer 1T-MoS_2_ and 1T-MoS_2_H tuned by strain

**DOI:** 10.1039/c7ra10304b

**Published:** 2018-02-23

**Authors:** Wei Xu, Shiming Yan, Wen Qiao

**Affiliations:** College of Science, Henan University of Technology Zhengzhou 450001 P. R. China shimingsam@163.com

## Abstract

The magnetic properties of 1T-MoS_2_ and 1T-MoS_2_H subjected to equiaxial tensile strain are calculated using density functional theory. It is shown that in a strain-free state, 1T-MoS_2_ and 1T-MoS_2_H both exhibit magnetic behavior; as the strain increases, their magnetic properties show an increasing trend. This shows a significant difference from those of 2H-MoS_2_ and 2H-MoS_2_H. Based on Crystal Field Theory, the magnetic generation and variation of 1T-MoS_2_ and 1T-MoS_2_H are explained in this paper. The good tunable magnetic properties of 1T-MoS_2_ and 1T-MoS_2_H suggest that they could be applied as a spin injection source for spin electronics.

## Abstract 2

The magnetic properties of 1T-MoS_2_ and 1T-MoS_2_H subjected to equiaxial tensile strain are calculated using density functional theory.

## Graphical abstract

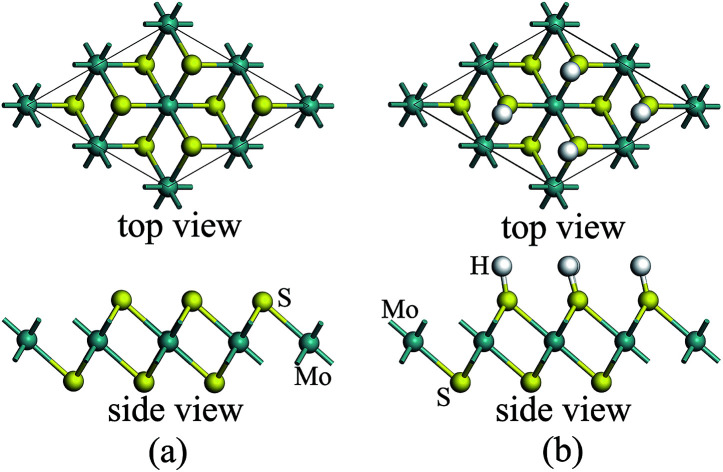

## 1. Introduction

Two-dimensional materials have attracted widespread attention because of their unique properties and many potential applications.^1–7^ In order to meet the needs of different applications, different methods are employed to tune their inherent nature.^8,9^

As a new type of two-dimensional material, MoS_2_ is widely studied in various fields because of its excellent electrical, optical and magnetic properties.^10–14^ Due to the different arrangement of atoms and the interlayer, there are mainly three different phases in block MoS_2_: 2H phase, 3R phase and 1T phase.^15^ Among them, the Mo atom in 2H-MoS_2_ has triangular prismatic coordination, and two S–Mo–S units form a unit cell; the Mo atom in 3R-MoS_2_ has triangular prismatic coordination, and three S–Mo–S units form a unit cell; the Mo atom in 1T-MoS_2_ has octahedral coordination, and each S–Mo–S monolayer forms a unit cell.^15^ In the three phases, the 2H phase is more general and stable, while the 1T phase and the 3R phase are metastable. They change to the 2H phase under the condition of heating. 1T-MoS_2_ is metallic, while the 2H phase and 3R phase are semiconductive.^16^ For monolayer MoS_2_, there are only two structures, namely the 2H phase and 1T phase.

In terms of magnetic properties, MoS_2_ may be widely used in spin electronic devices in the future. The study of such two-dimensional materials has been a considerable result, especially for the study of monolayer 2H-MoS_2_, revealing its unique properties in the monolayer structure.^2,17–23^ In general, two-dimensional materials can withstand greater strain.^24–28^ Relevant research shows that the degree of stretching monolayer MoS_2_ can be as high as 25%.^24,25,28^ Some theoretical calculations show that the application of strain to a two-dimensional transition metal disulfide can tune its magnetism. The application of strain has become an effective means of tuning the properties of two-dimensional materials.^29,30^ Existing studies have shown that the hydrogenation of the 2H-MoS_2_ can achieve the transition of non-ferromagnetic state to ferromagnetic state after applying strain.^17^ For the magnetism of 1T-MoS_2_, there is still a lack of research.

In this work, we calculated the magnetic properties of monolayer 1T-MoS_2_ and hydrogenated 1T-MoS_2_, and finally obtained a good linear variation of the magnetic properties with strain.

## 2. Details of calculation

For atomic hydrogenation onto monolayer 1T-MoS_2_, different positions, such as hole, S top, and Mo top, can be considered.^17^ However, previous studies have shown that the crystal structure is the most stable when the H atom adsorbed at the top of S atom.^17^ In this study, the H atom was only chemically adsorbed onto the S top site from one side of the monolayer 1T-MoS_2_ (denoted as 1T-MoS_2_H).

**Fig. 1:**
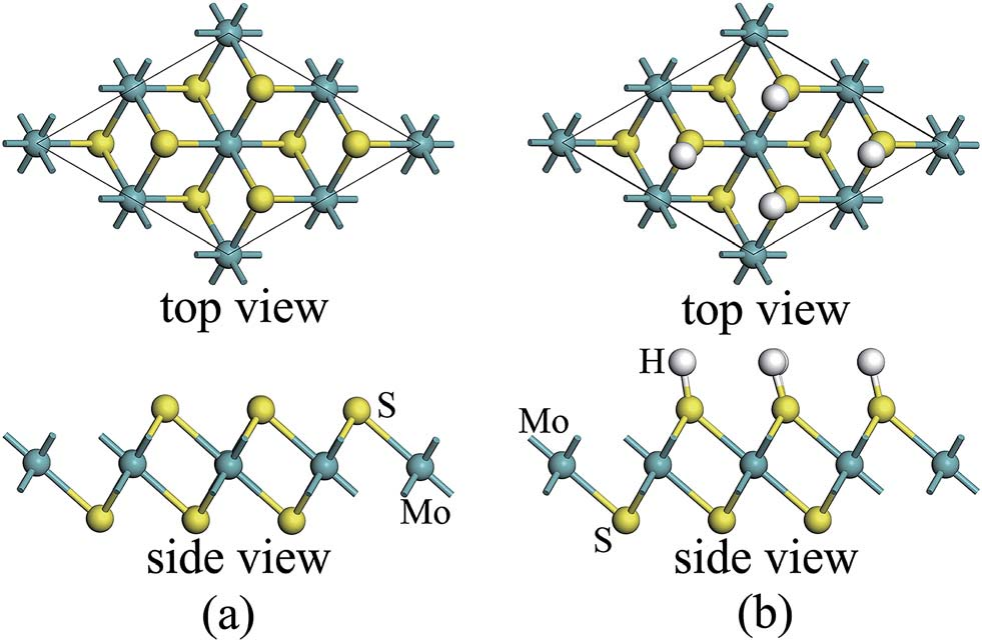
1T phase monolayer MoS_2_ model (a) without and (b) with hydrogen adsorption.

In the magnetic calculation, we selected 2 × 2 × 1 supercell. To eliminate the interaction between the layers, a vacuum layer being 10 Å thickness was provided in a direction that is perpendicular to the plane of the single layer MoS_2_. The structures of single layer 1T-MoS_2_ and 1T-MoS_2_H are shown in Fig. 1(a) and (b). Our density functional theory (DFT) calculations were performed by adopting the generalized gradient approximation (GGA) of the PBE functional for the exchange correlation potential.^31^ The electron wave function was expanded in a plane wave basis set with an energy cutoff of 550 eV. In the Brillouin zone, the *k*-point is taken as 10 × 10 × 1 and the integral of the Brillouin zone is calculated using the Monkhorst–Pack method.

## 3. Results and discussion

### 3.1 Atomic magnetic moment

**Fig. 2:**
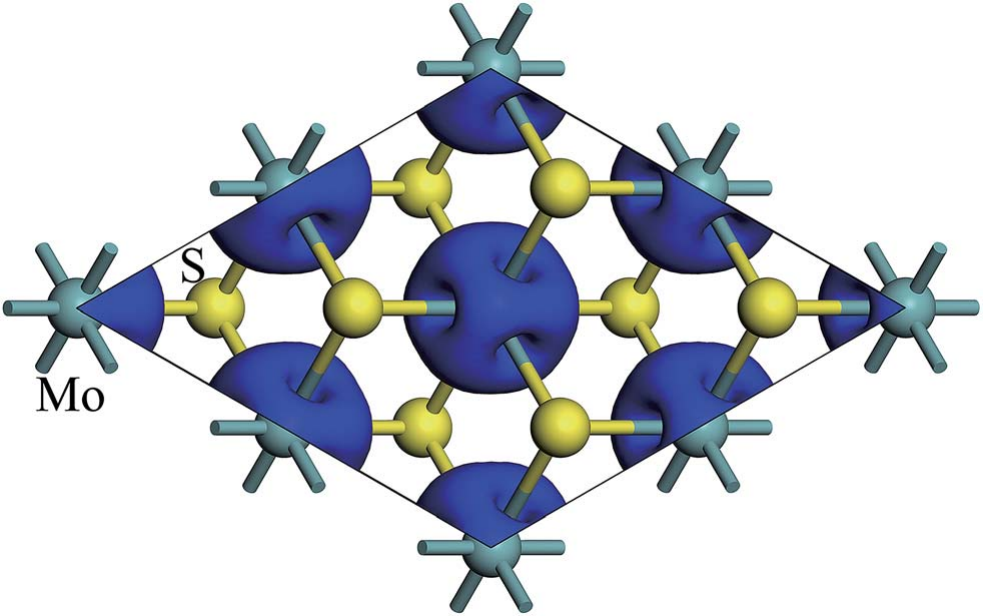
Contour plots of the spin density of monolayer 1T-MoS_2_ at tensile strain of 0%.

The spin density of 1T-MoS_2_ at 0% tensile strain is shown in Fig. 2. It can be seen from the figure that the spin density results mainly from Mo atoms, and the contribution of S atoms is relatively small. Based on the above analysis, it is concluded that the spin polarization state of 1T-MoS_2_ is dominated by Mo atoms and the contribution from other atoms is negligible. In absence of strain, spontaneous spin polarization has been observed in 1T-MoS_2_, which is consistent with previous studies.^32^

**Fig. 3:**
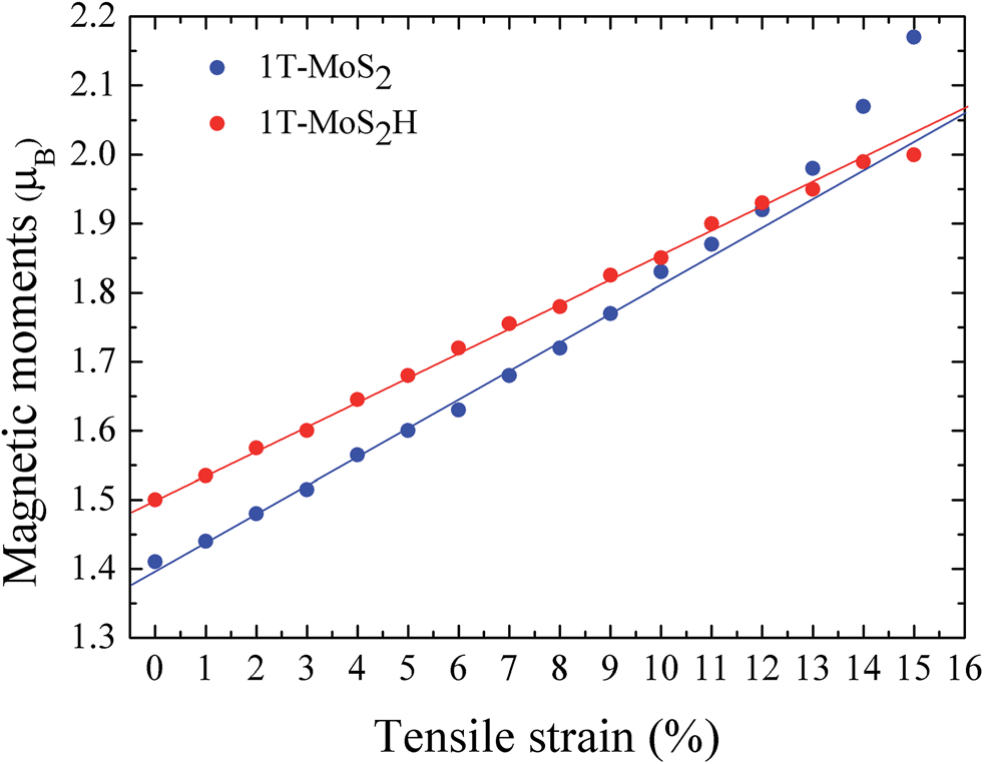
The function of magnetic moments of Mo atom in 1T-MoS_2_ and 1T-MoS_2_H as tensile strain. The blue and red points correspond to 1T-MoS_2_ and 1T-MoS_2_H respectively.

The magnetic moments are calculated with every 1% of the strain from 0% to 15%. The magnetic moments change as shown in Fig. 3. The magnetic moments of Mo atom in 1T-MoS_2_ at 0%, 3%, 6%, 9% and 12% tensile strain are 1.41 *μ*_B_, 1.52 *μ*_B_, 1.63 *μ*_B_, 1.77 *μ*_B_ and 1.92 *μ*_B_ respectively. It can be seen from the Fig. 3 that, with the strain increasing from 0% to 10%, the magnetic moments of Mo atoms in monolayer 1T-MoS_2_ linearly increase. When the strains are larger than 10%, the magnetic moments acceleratively increase.

Previous studies have shown that 2H-MoS_2_ is not magnetic, and even if the strain increases, its magnetism does not change.^26,33^ Here, 1T-MoS_2_ exhibits a distinctly different nature from 2H-MoS_2_.

**Fig. 4:**
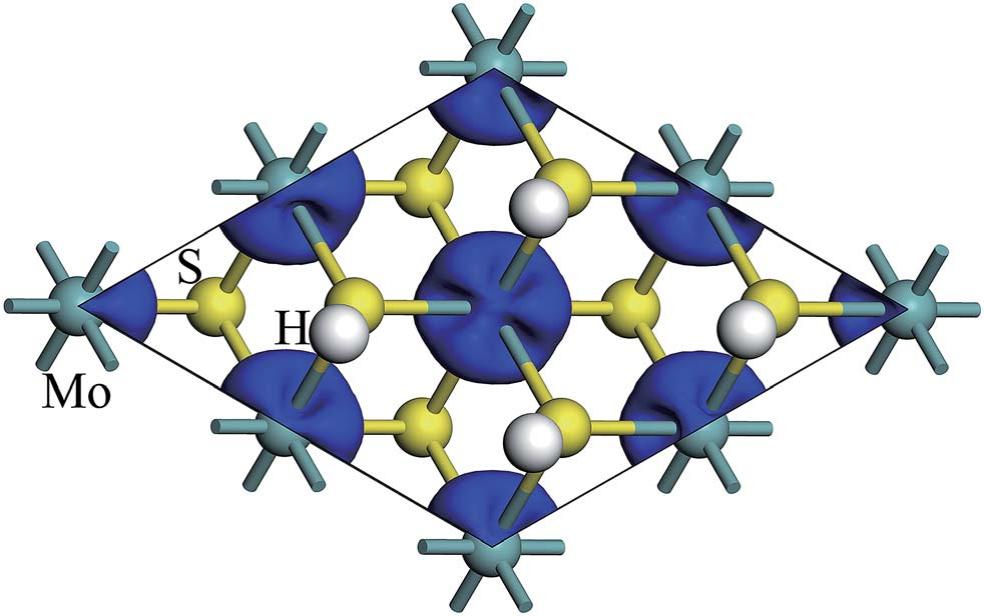
Contour plots of the spin density of monolayer 1T-MoS_2_H at tensile strain of 0%.

The spin density of 1T-MoS_2_H at 0% tensile strain is shown in the Fig. 4. Similar to 1T-MoS_2_, the spin polarization state is dominated by Mo atoms. Spin polarization exhibits when there is no strain, and gradually increases with enhancement of strain.

The magnetic moments are calculated with every 1% of the strain from 0% to 15%. The magnetic moments change as shown in Fig. 3. The magnetic moments of Mo atom in 1T-MoS_2_H at 0%, 3%, 6%, 9% and 12% tensile strain are 1.50 *μ*_B_, 1.60 *μ*_B_, 1.72 *μ*_B_, 1.83 *μ*_B_ and 1.93 *μ*_B_. It can also be seen that, with the strain increasing, the magnetic moments of Mo atom in monolayer 1T-MoS_2_H linearly increase. The coefficient of correlation of linear fitting is *R*^2^ = 0.9982, which indicates that the magnetic moments of the atom are very linear with the change of strain.

Shi *et al.* researched the magnetism of monolayer 2H-MoS_2_H.^17^ When there is no strain, the monolayer 2H-MoS_2_H has no magnetism. With the strain increasing, the magnetism of the monolayer 2H-MoS_2_H first increases and then decreases. Here, 1T phase and 2H phase also show a different trend.

From Fig. 3, comparing the changes of the magnetic moments of 1T-MoS_2_ and 1T-MoS_2_H, it can be seen that the magnetic moments of Mo atoms in hydrogen adsorption are larger than those without hydrogen adsorption when the strain is below 10%. For 2H phase, hydrogen adsorption also promotes the system to increase the magnetic moments of Mo atoms.^17^

### 3.2 Formation energy, phonon spectrum and DFT band structures

The formation energy of a hydrogen atom absorbed onto the S top site of monolayer 1T-MoS_2_ from one side is calculated as

**Fig. 5:**
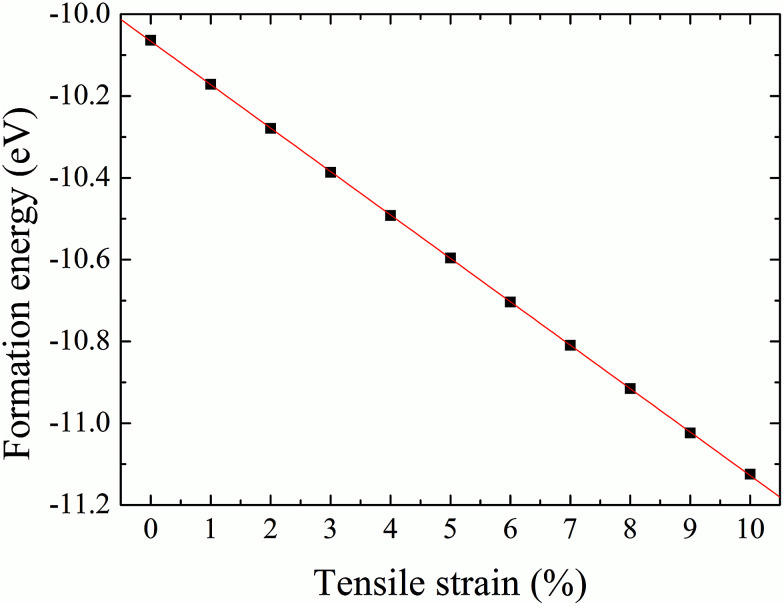
Formation energy of hydrogenation on monolayer 1T-MoS_2_ from one side as a function of tensile strain.

**Fig. 6:**
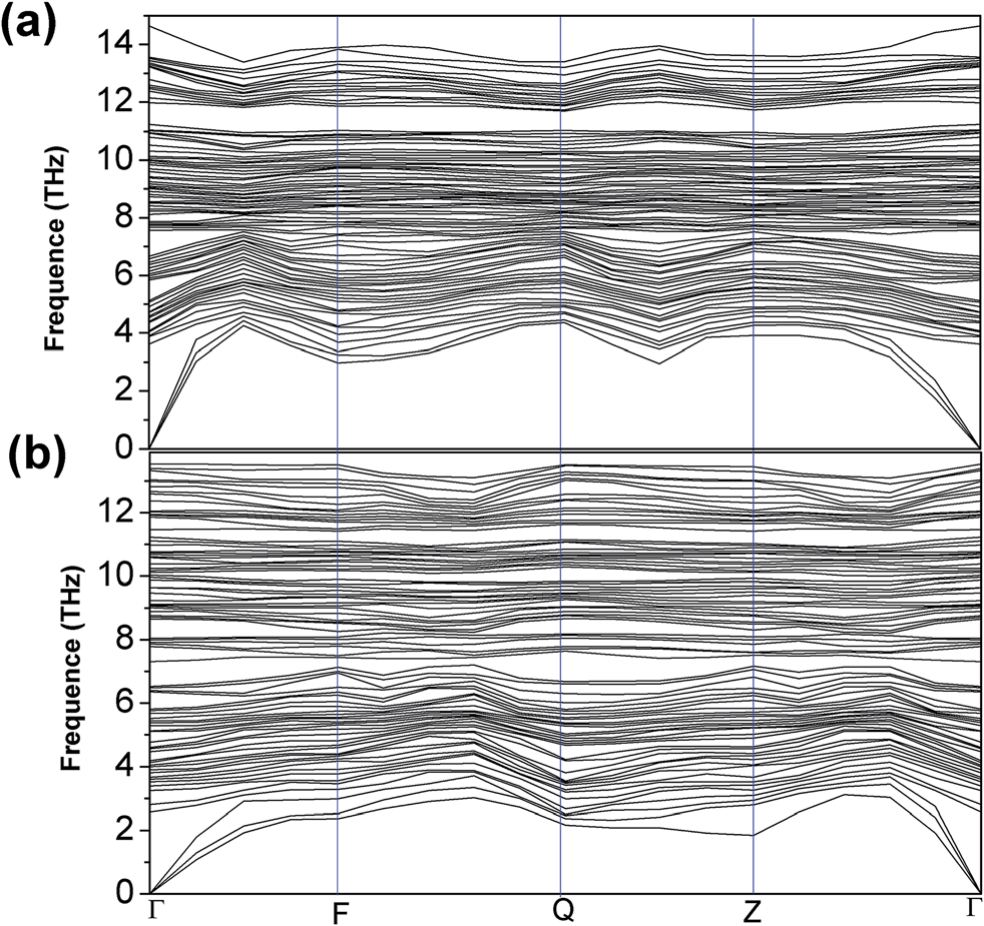
The calculated phonon spectra of 1T-MoS_2_ (a) and 1T-MoS_2_H (b).

**Fig. 7:**
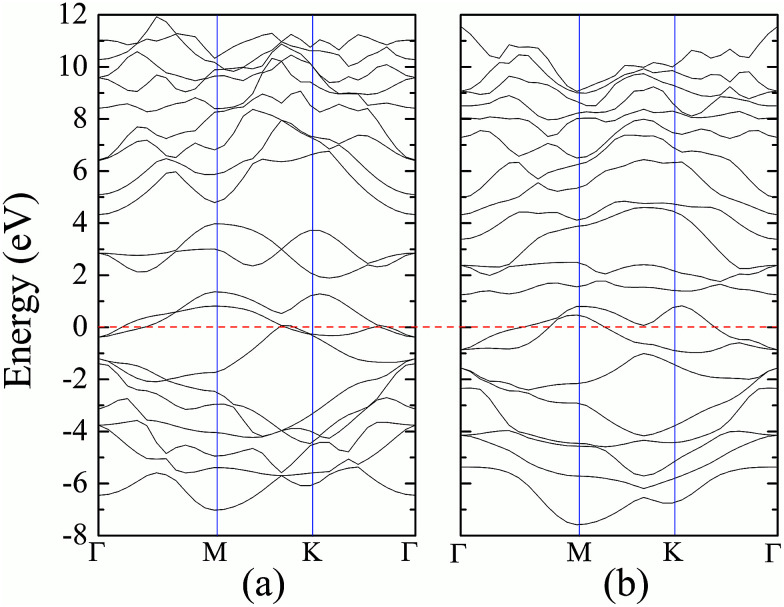
DFT band structures of 1T phase monolayer MoS_2_ (a) without and (b) with hydrogen adsorption.

1 *E*_a_ = *E*_tot_(MoS_2_H) − *E*_tot_(MoS_2_) − *E*(H),

where *E*_tot_(MoS_2_H) and *E*_tot_(MoS_2_) are the total energy of monolayer MoS_2_ when there is H atom adsorption and no H atom adsorption respectively.^17^*E*(H) is the energy of the H atom. The changes of formation energy with strain can be shown in Fig. 5. It can be seen that the 1T-MoS_2_H becomes more and more favorable as the tensile strain increases. The thermodynamic stabilities of 1T-MoS_2_ and 1T-MoS_2_H are also evaluated with phonon spectra calculation. Fig. 6 displays the calculated phonon dispersions of the 1T-MoS_2_ and 1T-MoS_2_H. There is no any presence of imaginary-vibration mode, indicating that the structures of 1T-MoS_2_ and 1T-MoS_2_H are dynamically stable. The calculated band structures of monolayer 1T-MoS_2_ and 1T-MoS_2_H are plotted in Fig. 7(a) and (b) respectively. No band gap at Fermi level was observed in the band structures, indicating that 1T-MoS_2_ and 1T-MoS_2_H are both metallic.

### 3.3 Density of states

**Fig. 8:**
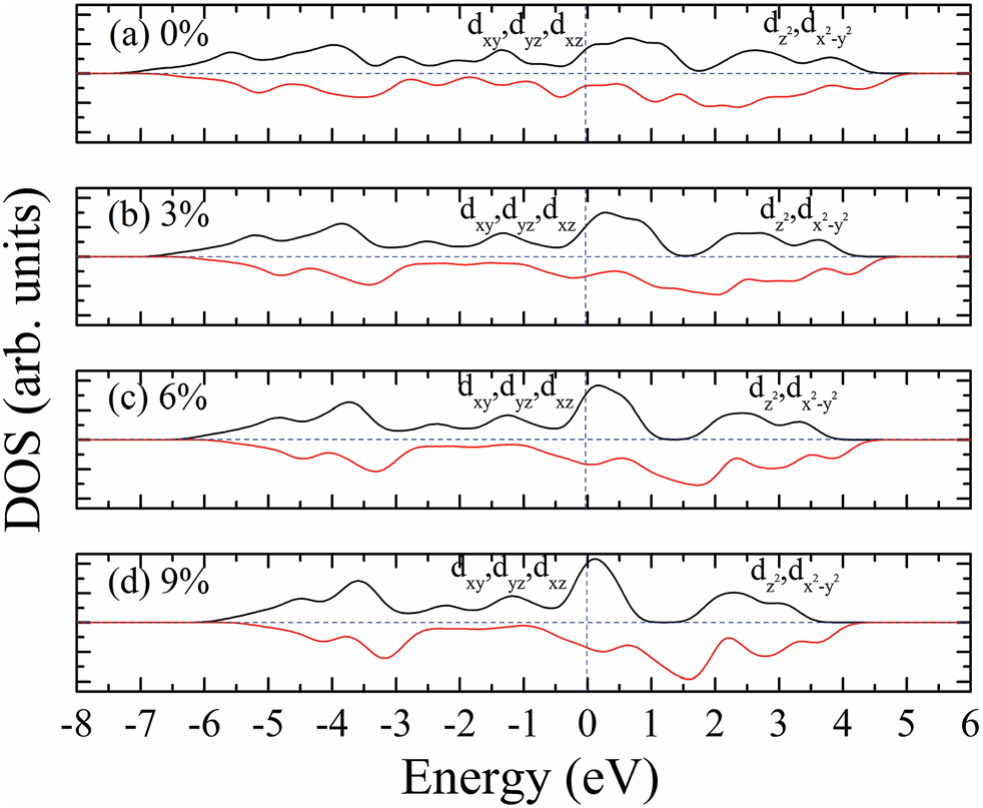
Projected density of states of Mo d orbitals for monolayer 1T-MoS_2_ at different tensile strain of (a) 0%, (b) 3%, (c) 6%, and (d) 9%, respectively. The black and red curves correspond to spin-up and spin-down components, respectively. The Fermi level is set to be zero.

According to our calculated spin density and magnetic moments, the 4d orbital electrons of Mo atoms dominate in the magnetic properties. The density of states of 4d orbital electrons of Mo atoms in 1T-MoS_2_ under different tensile strain is plotted in Fig. 8. As shown in Fig. 8(a), it can be seen that the spin-up and spin-down energy states of 4d electrons of Mo atoms are clearly cleaved for the monolayer 1T-MoS_2_. This suggests that Mo atoms in 1T-MoS_2_ has spin-unpaired 4d electrons and magnetic moments. We could see clearly from the density of states that 1T-MoS_2_ is no band gap and show a metallic property, which is consistent with the results reported in the ref. 16 and 32.

As the tensile strain increases, the spin-up and spin-down components move on opposite energy components, respectively, resulting in an increase in the net residual magnetic moments, as shown in Fig. 8. Spin polarization increases with strain.

**Fig. 9:**
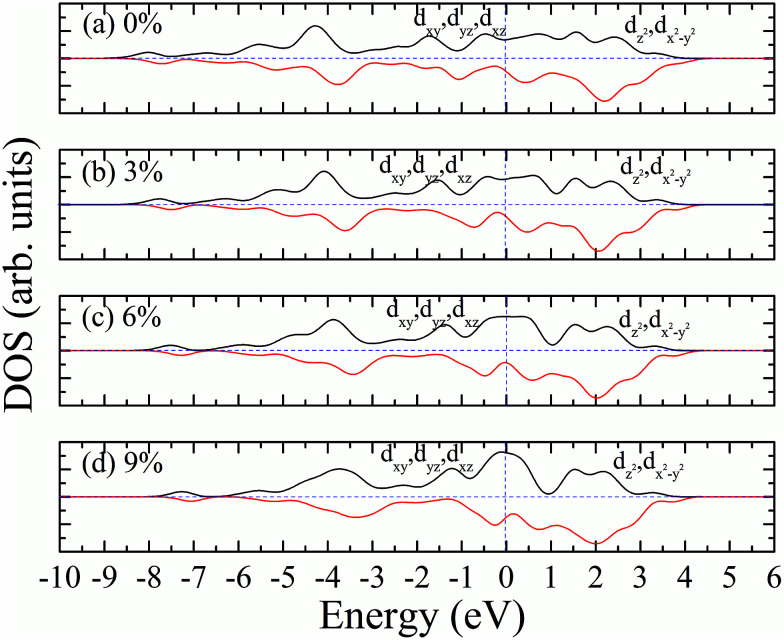
Projected density of states of Mo d orbitals for monolayer 1T-MoS_2_H at different tensile strain of (a) 0%, (b) 3%, (c) 6%, and (d) 9%, respectively. The black and red curves correspond to spin-up and spin-down components, respectively. The Fermi level is set to be zero.

Similarly, we calculated the electron energy density of states of the 4d orbit of Mo atoms in monolayer 1T-MoS_2_H, as shown in Fig. 9. It can be seen from the figure that the variation trend of spin-splitting and magnetic moments are consistent with those of hydrogen-free adsorption.

### 3.4 Magnetic state

**Fig. 10:**
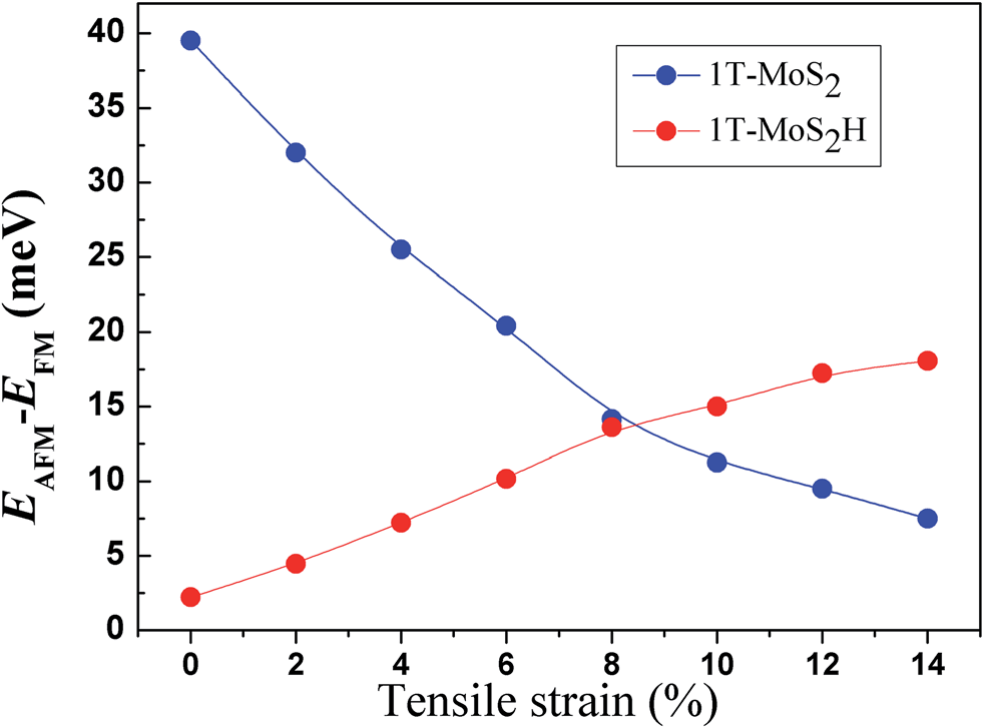
Strain dependence of the energy difference per unit cell between FM and AFM coupling.

The spin-ordering dependence on strain in 1T-MoS_2_ and 1T-MoS_2_H is explored. The energy difference, Δ*E* = *E*_AFM_ − *E*_FM_, per unit cell as a function of tensile strain is presented in Fig. 10. The energy of ferromagnetism (FM) is larger than that of antiferromagnetism (AFM) for both 1T-MoS_2_ and 1T-MoS_2_H with different strains, indicating that the AFM coupling is favored over the FM coupling. With increasing strains, energy difference of AFM and FM increase for 1T-MoS_2_H, but decrease for 1T-MoS_2_. This difference of trends of strain dependence spin-ordering could be ascribed to the modification of exchange interaction induced by H absorbing. Magnetic state for magnetic 2D compounds can be explained using through-bond coupling interaction and through-space coupling interaction.^29^ Through-bond coupling can induce ferromagnetic coupling, while through-space coupling can induce AFM coupling.^29^ For the 1T-MoS_2_ and 1T-MoS_2_H, spin-ordering dependence on strain could be the result of competitive effect between the through-bond interaction and the through-space interaction.

### 3.5 Discussion

**Fig. 11:**
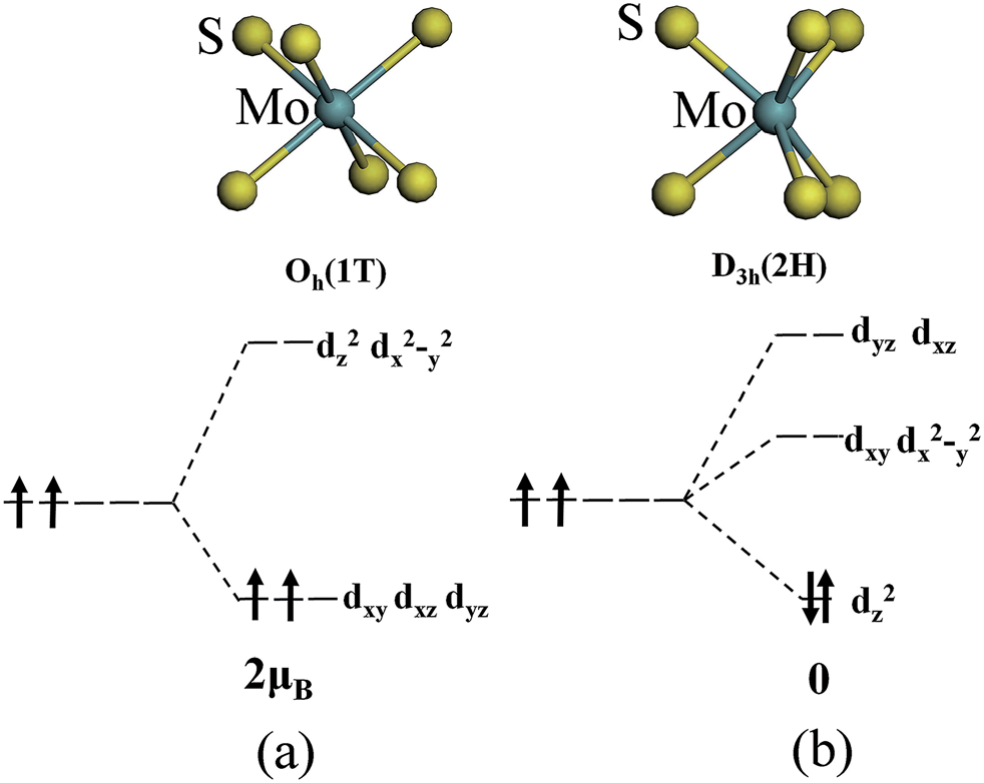
The occupation of electrons in Mo 4d orbits under the crystal fields of (a) 1T phase and (b) 2H phase.

The magnetism of 1T-MoS_2_ can be explained with the Crystal Field Theory. As shown in Fig. 11(a),^32^ in the monolayer 1T-MoS_2_, the Mo atom is located at the center of the antilayer prism crystal field, and the state of 4d orbital electrons is split into d_*x*^2^−*y*^2^_/d_*z*^2^_, d_*yz*_/d_*xz*_/d_*xy*_, where d_*yz*_, d_*xz*_ and d_*xy*_ compose triple degenerate state for the ground state.^34,35^ According to the reference, the energy ranges for these energy levels are also labeled in Fig. 8.^36^ Electrons of Mo atom arranged for 1s^2^2s^2^2p^6^3s^2^3p^6^3d^10^4s^2^4d^5^5s^1^. The valence state of Mo atom in 1T-MoS_2_ is +4, and there are two remaining electrons in the outermost layer of Mo atom. According to Hunt rule and lowest energy principle, in order to ensure that the energy of the system is as small as possible, the two electrons of 4d orbits tend to be arranged in the same direction and randomly occupy two orbits of the ground state. Therefore, there is a net magnetic moment 2 *μ*_B_ of Mo atom in 1T-MoS_2_. Fig. 8 shows that the d_*xy*_/d_*yz*_/d_*xz*_ degenerate ground energy level is partially filled, whereas the d_*z*^2^_/d_*x*^2^−*y*^2^_ degenerate energy level is higher than Fermi level and is not filled. The calculation results are consistent with the theory.

Our calculations show that the Mo atoms in 1T-MoS_2_ have a net residual magnetic moment of 1.41 *μ*_B_, which is slightly lower than that given by Crystal Field Theory. There are two possible reasons for this. One possible reason is that 1T-MoS_2_ is metallic, where electrons are in a free state, so that electrons do not completely occupy the 4d orbits of Mo atoms. Another possible reason is that Crystal Field Theory only considers the interaction between the Mo atom and the nearest neighbor S atoms. However, in the case where the interaction with the atoms in the sub-nearest neighbor and the greater position is taken into account, the cleavage of energy level of the Mo atom might be more complex.

As shown in Fig. 11(b),^32^ the Mo atom in the 2H-MoS_2_ is located at the center of the triangular prism crystal field, and the 4d orbital electron state is split into d_*x*^2^−*y*^2^_/d_*xy*_, d_*yz*_/d_*xz*_ and d_*z*^2^_ under the action of the triangular prism field, where d_*z*^2^_ state is ground state.^34,35^ The valence state of Mo atom in 2H-MoS_2_ is +4, and there are two remaining electrons in the outermost layer of Mo atom. Since the Coulomb interaction energy of the 4d orbital electrons is smaller than that of the crystal field splitting energy, the two 4d orbital electrons tend to occupy the lowest energy ground state according to lowest energy principle. According to Pauli exclusion principle, two 4d orbital electrons are arranged in the reverse direction of spin, and there is no spin-unpaired electrons. Thus, there is no net residual magnetic moment in 2H-MoS_2_, only showing the diamagnetism. The above discussion illustrates the difference in the magnetic properties of 1T-MoS_2_ and 2H-MoS_2_ when no strain is applied.

In the following, the magnetic variation of 1T-MoS_2_ is discussed when strain increases. Strain-induced magnetism in transition metal dichalcogenide (TMD) monolayers has been ascribed to the competition between covalent-bond and ionic-bond interactions.^29,30^ For the most 4d/5d transition-metal dichalcogenides, due to the strong ligand field, the magnetic moment of 4d/5d transition-metal is quenched. When the structure is under a finite tensile strain, the covalent-bond reduce, and the ionic-bond enhance, inducing the existence of magnetic moment for 4d/5d transition-metal.^29,30^ For the 1T-MoS_2_, this mechanism could also play an important role. With the increase of tensile strain, the number of electrons involved in the covalent-bond decreases and the number of electrons located at Mo atoms increases, enhancing the magnetic moment of Mo atoms.

In addition, the magnetism dependence on strain could also be affected by crystal field. For the 1T-MoS_2_, due to the special crystal field around Mo ions formed by the nearest neighbor ions, there could be net residual magnetic moment of 2 *μ*_B_.^32^ Actually, under the effect of sub-nearest neighbor and the greater position ions, the magnetic moment will slightly decrease and be lower than 2 *μ*_B_. The equiaxial tensile strain do not modify the symmetry of crystal field formed by nearest neighbor ions. However, increase of tensile strain can reduce the strength of the crystal field of sub-nearest neighbor and the greater position ions, decreasing its effect on magnetism and leading to increase of magnetic moment of Mo atom in 1T-MoS_2_.

**Fig. 12:**
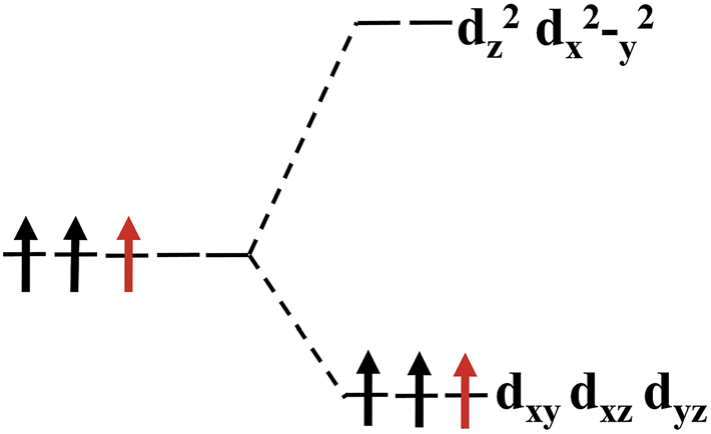
The arrangement of the extra electrons in the energy level.

Our calculations show that, when the strain is below 10%, the 4d orbital electrons of Mo atom in the hydrogenated MoS_2_ is larger than that of the MoS_2_ which is not hydrogenated. This suggests that the hydrogenation process occurs where the charge is transferred, and hydrogenation can contribute to the addition of additional electrons to the 4d ground state of Mo atom. By comparing Fig. 8 with 9, it can be found that the energy peak of the d_*xy*_/d_*yz*_/d_*xz*_ degenerate state shifts to the left and the Fermi level shifts to the right of this peak, which also indicates that hydrogenation causes more electrons to participate in the filling. This is consistent with the foregoing. It is assumed that there is one extra electron that enters the 4d orbits of Mo atom to fill. According to Hunt rules and lowest energy principle, the additional electron tends to be in the same direction as the arranged electrons, as shown in Fig. 12. Therefore, according to Crystal Field Theory, Mo atom in monolayer 1T-MoS_2_H has 3 *μ*_B_ net residual magnetic moments. Likewise, the calculated value here is slightly lower than the theoretical value given by Crystal Field Theory, whose reason is the same as that when there is no hydrogen adsorption. When the strain increases, the variation of the 1T-MoS_2_H electron arrangement can be explained in the same way with 1T-MoS_2_, and the difference is that the electrons occupying the 4d orbits in the hydrogenated MoS_2_ is more than that in the absence of hydrogen adsorption. Thus, compared with the hydrogen-free adsorption, the magnetic moment is larger, which is consistent with the calculated results.

When the strain is above 10%, the magnetic moments of 1T-MoS_2_ acceleratively increase, and ultimately exceed that of 1T-MoS_2_H with the strain increasing. This result can be ascribed to the different strength of covalent-bond for 1T-MoS_2_ and 1T-MoS_2_H with strain above 10%. Below 10%, the effect of crystal field dominates the magnetic moment, and the magnetic moments show linearly with the change of strain. Above 10%, the reduction of covalent-bond under strain could be an accelerated process, and the competition between the covalent-bond and the ionic-bond interactions dominates the magnetic moments. The accelerated increase of magnetic moment with increasing strain was also be found in other TMDs.^29,37^ Compared to 1T-MoS_2_H, covalent-bond of 1T-MoS_2_ could be more easy to reduce under large strain. Thus, the magnetic moments of 1T-MoS_2_ appear the accelerated increasing trend with strain above 10%.

## 4. Conclusions

We studied the monolayer 1T-MoS_2_ and 1T-MoS_2_H using density functional theory. The results show that there is magnetism in 1T-MoS_2_ at zero strain, and magnetic moments of 1T-MoS_2_ mainly result from Mo atoms. As the tensile strain increases, the magnetic moments of Mo atom gradually increase, and the magnetism of 1T-MoS_2_ enhances. For the 1T-MoS_2_H, when the strain is below 10%, the magnetic moments are larger than 1T-MoS_2_, while the change trend is consist with that without hydrogen adsorption. When the strain is above 10%, the magnetic moments of 1T-MoS_2_ appear the accelerated increasing trend. The tunable good magnetic properties of 1T-MoS_2_ and 1T-MoS_2_H suggest that they would be applied as a spin injection source for spin electronics.

## Conflicts of interest

There are no conflicts to declare.

## Supplementary Material
